# Bayley III in Vietnamese children: lessons for cross-cultural comparisons

**DOI:** 10.12688/wellcomeopenres.15282.1

**Published:** 2019-06-19

**Authors:** Luning Sun, Saraswathy Sabanathan, Pham Ngoc Thanh, Anh Kim, To Thi Mai Doa, C. Louise Thwaites, H. Rogier van Doorn, Bridget Wills

**Affiliations:** 1 The Psychometrics Centre, University of Cambridge, Cambridge, CB2 1AG, UK; 2 Oxford University Clinical Research Unit, Hospital for Tropical Diseases, 764 Vo Van Kiet, Vietnam; 3 Paediatric Neurology, Cambridge University Hospital Foundation Trust, Hills Rd, Cambridge, CB2 0QQ, UK; 4 Centre for Tropical Medicine and Global Health, University of Oxford, Nuffield Department of Medicine Research Building, Oxford, OX3 7FZ, UK

**Keywords:** Bayley III, child development, adaptation, validation, Vietnam

## Abstract

**Background:** There are limited psychometric reports of construct validity following adaptation of the Bayley Scales of Infant and Toddler Development 3 ^rd^ edition (Bayley III). This paper aims to demonstrate a process of assessing reliability, validity, and gender equivalence of the adapted tool for Vietnamese children.

**Methods:** We evaluated cognitive, fine motor, gross motor, expressive communication and receptive communication subtests of the adapted tool in 267 healthy urban Vietnamese children. Subsets of participants were used to evaluate inter-observer and test-retest reliability. Confirmatory factor analysis (CFA) was carried out to evaluate construct validity and measurement invariance between genders.

**Results:** The adaptation demonstrated good inter-observer and test-retest reliability. CFA indicated that a construct representing a single underlying factor showed the best fit, although relationships between the observed scores and the latent traits underlying the scores varied between age groups. Within age groups, relationships between observed scores and these factors were not significantly influenced by gender.

**Conclusions:** The Vietnamese Bayley III demonstrated good internal consistency and reliability. A latent structure with one general factor and additional residual correlations that change with age is supported by the theoretical understanding of child development. This is the first study to demonstrate gender invariance by age group. This adaptation is suitable for further research studies in urban Vietnamese children, but further work is needed to extend its applicability more broadly across Vietnam.

## Introduction

Over the last 25 years, an unprecedented reduction in under-five mortality has been achieved under the Millennium Development Goals ^1^. Following adoption of the Sustainable Development Goals in 2015 ^2^, there is now increased focus on children’s early development. Valid and reliable child development assessment tools (CDATs) are vital to evaluate needs and assess outcomes of intervention programmes.

The Bayley Scales of Infant and Toddler Development, Third Edition (Bayley III) ^3^, is widely used internationally to evaluate early child development. The tool assesses five domains: a) cognitive (91 items), b) language (receptive language, 49 items; expressive language, 48 items), c) motor (fine motor, 66 items; gross motor, 72 items), d) socio-emotional (35 items) plus e) adaptive behaviour (241 items). It was standardised on a cohort of 1700 US children, stratified by age, sex, parental education, race and geographic region (US norms) ^3^. Raw scores are converted to scale scores and then to composite scores, which are used to determine the child's performance compared with these US norms. Worldwide these norms are commonly used as the reference population ^4– 6^.

The Bayley III was formulated on the principle that it measures underlying traits or latent factors. Confirmatory factor analysis (CFA) was used to demonstrate construct validity by evaluating relationships between test scores and different underlying traits/factors. The authors concluded that the test scores best modeled three underlying traits – motor, language and cognitive factors. This was evaluated on the total standardization sample of 1700 children, with the sample split into 4 age groups of 300–600 children per group. The manual does not explain the rationale for selecting the age groups ^7^.

When an assessment tool is adapted for use in another cultural context, it is important to demonstrate that the relationship of the observed scores to the underlying hypothesised traits (i.e. factors) is comparable to the original. This process of establishing construct equivalence of the adapted tool ensures validity of the test in the new setting. Van de Vijver describes increasing levels of equivalence ^8^, culminating in full score equivalence, where the relationships between the test scores and their construct scales (i.e. the theoretical framework) have both the same measurement unit and origins ^9, 10^. This is the only situation where scores have the same distribution in both groups or cultures compared, making it appropriate to use score means for analyses of variance and t-tests for group comparisons ^11^.

Measurement invariance analysis can be used to assess construct equivalence between and within populations through a step-wise increase in model constraints. The best fitting model of construct validity is first compared between groups at baseline (i.e. configural) level, and then specific parameters in the models are increasingly constrained to assess invariance at different levels: metric invariance; scalar invariance, which permits construct-level comparisons between groups; ^12^ and finally strict invariance, although this is rarely achieved ^13^. It is accepted that for cross-cultural comparisons scalar invariance is sufficient ^12, 14^. By contrast, for within population comparisons (such as by gender) validity at the metric level is acceptable, implying that respondents from both groups understand the test and respond in similar fashion ^8, 15^. Standardisation data is not publicly available, so evaluation is limited to determining whether the same original construct structure holds true in the new population.

For within culture or between population comparisons, differences in scores between groups may be biased by group membership rather than indicating a true difference between the groups. For example, there have been consistent gender differences in pre-term neonatal outcomes in studies which have used the Bayley scales in the US ^16^ and Sweden ^17^. However, there is no data on whether the Bayley III is gender invariant, i.e. whether the scores and their relationship to the underlying constructs being assessed are the same, irrespective of gender, within the same population. Gender differences in behaviour between cultures are well described ^14^, and an adapted tool may demonstrate different effects of gender on the theoretical constructs.

Establishing robust psychometric properties for an adapted CDAT is important to allow meaningful interpretation of the data collected using the tool. Here we describe in detail the processes we used to adapt the Bayley III for use in an urban setting in Vietnam, as well as our assessments of reliability, construct validity and gender equivalence of the adapted tool in a group of healthy Vietnamese children. This undertaking was part of a wider programme of work focussed on evaluating neurodevelopmental outcomes following severe hand foot and mouth disease in Vietnam.

## Methods

### Adaptation of the Bayley III tool

In summary, adaption consisted of translation, evaluation of cultural modifications through the group’s experience, back-translation, and implementation of the test in a group of volunteers (pilot testing) resulting in further modifications (see extended data ^18^).

After direct translation into Vietnamese by 3 psychologists, we adapted the cognitive, language and motor domains of the Bayley III in line with guidance from the International Test Commission ^19^, and recommendations from publications on reducing cultural bias ( Table 1) ^20– 23^. Six Vietnamese psychologists and one special needs teacher reviewed the direct translation for ambiguity, following which a post-doctoral language expert gave further advice ^24^. An independent Vietnamese bio-scientist then carried out a direct back-translation, and any discrepancies were reviewed and amended by another two independent bio-scientists who had lived for 2 or more years with their children in the US. After pilot testing on 30 children a final version was created. Additionally, 18 children from the pilot testing had their assessments videoed. These eighteen videos were used to assess inter-observer reliability.

**Table 1.** Types of bias and strategies used to limit these in the Vietnamese adaptation of Bayley III.

Type of Bias	Strategies used in Vietnamese adaptation of Bayley III
Construct bias – two different meanings in two different cultures	Use of informants with expertise in local culture and language to review the items and evaluate whether the adaptation still evaluates the same domain as the original.
Method bias – language dialect differences	Extensive training of the staff administering the Bayley III and creation of standardised procedures
Lack of familiarity with a stimulus	Example: all children were introduced to the ‘bear’ or ‘block’ in the same way at the same time in the assessment.
Scoring responses from children consistently	Detailed manual/protocol for administration, scoring, and interpretation. Staff training. Use of test-retest strategies.
Item bias	Qualitative judgments by local experts on specific items that are inappropriate for Vietnam, e,g washing machine item or picture with snow.

Pearson Education Inc. granted the study team a licence for the translation and adaptation. The seven assessors underwent 6 months of training.

### Study sites and selection of participants

The children in this study were enrolled as controls for a cohort study of enterovirus 71 infection and neurological development. The inclusion criteria were; children aged less than 4 years from District 8 HCMC. The exclusion criteria were; history of chronic severe illness (e.g congenital cardiac disease, epilepsy), ex-premature (born <37 weeks gestation), prior intensive care admission, or known developmental delay. Potential participants attending one of three specific kindergartens in District 8 in Ho Chi Minh City (HCMC), were approached about the study. Participants were also recruited from a long-term birth cohort run as a collaboration between Oxford University Clinical Research Unit (OUCRU) and Hung Vuong Government Maternity Hospital ^25^. Mothers of these infants were approached about the Bayley III evaluation when they attended routine study visits at 4, 9, 12 or 18 months after birth. Additional recruitment occurred at three government primary care clinics, administered by the Preventive Medicine Centre in District 8, HCMC, on routine immunisation days. Recruitment from all sites occurred between September 2013–2014. Any child deemed to have a developmental problem was excluded from the study. Children were tested up to three times in 18 months resulting in a total of 476 assessments. Evaluation of construct validity used the first assessment data (N=267).

### Administration of the Bayley III

Following written informed consent, the parent/guardians of study participants were given an appointment for Bayley III testing, which was performed in a quiet private room either at the recruitment site or at OUCRU. Administration followed the original Bayley manual guidance. Cognitive, receptive and expressive language, fine and gross motor subtests were administered to each child on two occasions six months apart by the trained study staff. The child’s age in months determined the start test item for each subtest. A standard case report form was used to record demographic and socio-economic data (see extended data ^18^).

### Ethical considerations

Ethical approval was granted by the Oxford Tropical Research Ethics Committee (OxTREC approval number: 33-12) and the Institutional Review Boards of the Hospital for Tropical Diseases and Children’s Hospital 1, HCMC, (ND1 approval number: CS/N1/2012/038). The overall study programme was registered at ClinicalTrials.gov on 19 February 2014 (NCT02066714).

### Evaluation and statistical methodology

Reliability: Internal consistency was evaluated using Cronbach’s alpha (acceptable values 0.7–0.9) ^26^. All seven assessors independently scored the same 18 videos (pilot testing) for inter-observer reliability. This was assessed using intra-class correlation (ICC). Test-retest reliability was assessed with Pearson’s correlation, carried out in a convenience sample of study participants where the parents/guardians were willing to return within 2 weeks.

Construct validity: CFA was used to determine the underlying structure using data from 267 children. Due to the limited sample size, we assessed three age groups 0–12 months (N=86), 13–24 months (N=110), and 25–42 months (N=71). A single factor (general neurodevelopment) was specified in the CFA. If the model fit was not acceptable, modification indices were examined to identify areas of model misfit. Particularly, it highlighted items which shared common variance in addition to the underlying factor, suggesting correlated residuals that could better explain the observed pattern in the scores. Including these correlated residuals in the model would improve model fit ^27^.

Measurement invariance between genders: This was carried out using multiple group confirmatory factor analysis (MGCFA) for each age group, using the pre-specified best model from CFA. MGCFA progressively places constraints onto the model and if the model continues to show adequate fit, measurement invariance at this level is demonstrated.

Goodness of fit indices: The following measures of overall model fit were used, each with standard indices for goodness of fit: root mean square error of approximation (RMSEA, acceptable fit <0.08, good fit <0.05); ^28^ the comparative fit index (CFI, acceptable fit >0.90, good fit >0.95); ^29^ the Tucker-Lewis index (TLI, acceptable fit >0.90, good fit >0.95) ^29^. A p-value of 0.05 was taken to be significant in all analyses. Akaike’s information criterion (AIC) and Bayesian information criterion (BIC) were used to evaluate the trade-off between model fit and complexity of the model; a lower AIC or BIC value indicates a better fit when comparing models.

A combination of measures was used as the RMSEA may be negatively influenced by a small sample size and small degrees of freedom ^30^. The Chi square is positively influenced by the sample size, whereas TLI and CFI are less affected by sample size. A model was deemed to have good fit when the chi square was not significant, with CFI and TFI >0.95 and RMSEA <0.05. Additionally, an improvement in fit between comparative models was identified by a reduction in AIC and BIC. Measurement invariance was evaluated using nested models. A change in CFI between nested models of ≥ 0.01 identified a lack of invariance ^31^.

All statistical analyses were carried out in R version 3.2.1 ^32^. Package ICC version: 2.3.0 ^33^ was used to calculate the ICC estimates, which are based on mean squares obtained by applying analysis of variance models to the data. Lavaan package version: 0.5–23.1097 was used for the CFA and measurement invariance analyses ^32, 34, 35^.

## Results

### Characteristics of the study population

A total of 267 children aged 3–43 months were enrolled in the study between September 2013 and January 2014. Among this group, 191 children (72%) were recruited from the birth cohort, 54 (20%) from kindergartens, and 22 (8%) from the government primary care clinics. Table 2 compares the cohort to the publicly available Multiple Cluster Survey 2011 ^36^. Using Fisher’s exact test we identified significant differences in the proportions of stunted children (Odds Ratio (OR) 2.29, 95%CI 1.26-4.48, p-value=0.04) and levels of maternal education (no school/primary only OR 0.24, 95%CI 0.00-0.89, p-value < 0.01, secondary OR 0.64, 95%CI 0.50-0.83, p-value < 0.01 and higher education OR 2.62, 95%CI 2.00-3.43, p-value = <0.01) between the study and census data.

**Table 2.** Comparison of cohort to the Multiple Cluster Survey 2011 ^36^.

	Healthy Cohort Study Population			Multiple Cluster Survey 2011 ^36^			
				National prevalence of stunting N=3678			Urban stunting N=983
Study N=267	Male n=147	Female n=120	Both sexes n=267	Male N=1821	Female N=1751	Both sexes N=3678	Both Sexes N=112
**Age at enrolment in months** **Median (IQR)**	15.87 (16.16)	15.97 (12.16)	15.77 (14.25)				
**Z scores: length for age (all** **data) Mean (SD)**	-0.93 (1.45)	-0.76 (1.44)	-0.76 (1.64)				
**Stunted (<-2SD z scores:** **length for age according to** **WHO guidelines) ^37^**	30 (20%)	13 (11%)	43 (16%)	432 (23.7%)	378 (21.6%)	835 (22.7%)	112 (11.4%)
Maternal Education (rural and urban, both sexes). N=3678							
**No school or primary only** **(% of total)**	21 (14%)	22 (18%)	43 (16%)	865 (23.5%)			
**Secondary school (% of** **total)**	71 (48%)	55 (46%)	126 (47%)	2149 (58.4%)			
**Higher education (% of** **total)**	55 (37%)	43 (36%)	98 (37%)	664 (18.1%)			

### Reliability

Internal consistency of the domain subsets with all ages combined (N=476) was very good with Cronbach’s alpha 0.95 to 0.97 for each domain ( Table 3). When scores were analysed within individual age groups, acceptable consistency was maintained (Cronbach’s alpha >0.7), except for the fine motor domain at 18–24 months and for receptive language aged less than 12 months. Raw score ICC inter-observer variability was very good (>0.90) in all domains. Test-retest reliability was evaluated in between 25 and 29 children, according to the specific domains assessed, using Pearson’s correlation. Correlations of raw scores were high in all domains, with correlation coefficients ranging from 0.96 to 0.97 for all assessments.

**Table 3.** Internal consistency (using Cronbach’s alpha by age group and for all ages combined), test-retest reliability, and inter-observer reliability for the 7 assessors.

	INTERNAL CONSISTENCY (ICC) ^α^					TEST-RETEST RELIABILITY			INTER-OBSERVER RELIABILITY		
	≤12	>12 ≤18	>18 ≤24	>24 ≤43	All Ages	N	Days difference between tests ^#^	Pearson correlation	N	ICC	CI
**N**	119	113	95	149	476						
**Cognitive**	0.91	0.82	0.77	0.90	0.97	29	9 (2 to 31)	0.97 (0.94-0.99) **	20	0.99	0.99-0.99
**Receptive Language**	0.62	0.85	0.88	0.90	0.96	28	9 (2 to 30)	0.96 (0.95-0.99) **	21	0.978	0.96-0.99
**Expressive Language**	0.79	0.87	0.92	0.89	0.97	27	9 (3 to 30)	0.97 (0.94-0.99) **	18	0.97	0.94-0.99
**Fine Motor**	0.89	0.73	0.58	0.83	0.95	29	9 (2 to 31)	0.97 (0.96-0.99) **	19	0.99	0.98-0.99
**Gross Motor**	0.93	0.86	0.72	0.85	0.97	25	8.1 (3 to 16)	0.96 (0.91-0.98) **	20	0.99	0.97-0.99

^α^Repeated assessments included ^#^ Median (range). **p value<0.01.

ICC intraclass correlation. CI: 95% Confidence interval. N= no of cases.

### Construct validity and gender measurement invariance

We present here the CFA results for a general factor and measurement invariance by age group ( Table 4– Table 6)

**Table 4.** Confirmatory Factor Analysis of Vietnamese adaptation of Bayley III.

	Goodness-of-Fit Indices									
Model	X2	Df	X2/Df	p-value	AIC	BIC (Adjusted)	CFI	TLI	RMSEA (CI)	MI
Group 1 (N=86) Age >0 =<12 months										
Null Model	418.03	10	41.80							
Model 1	12.74	5	2.55	0.03	2079.60	2104.14 (2072.59)	0.98	0.96	0.13 (0.04-0.23)	Fine Motor ~~ Gross Motor, 7.96
Fine Motor ~~ Gross Motor	5.62	4	1.41	0.23	2074.28	2101.48 (2066.78)	1.00	0.99	0.07 (0.00-0.19)	
Group 2 (N=110) Age 12 =<24months										
Null Model	505.51	10	50.55							
Model 1	35.00	5	7.00	<0.001	2961.90	2988.91 (2957.31)	0.94	0.88	0.23 (0.16-0.31)	Expressive ~~ Receptive, 35.31
Expressive ~~ Receptive	2.24	4	0.56	0.69	2931.14	2960.85 (2926.09)	1.00	1.00	0.00 (0.00-0.11)	
Group 3 (N=71) Age >24<=43months										
Null Model	267.05	10	26.71							
Model 1	7.90	5	1.58	0.16	1969.20	1991.83 (1960.33)	0.99	0.98	0.09 (0.00-0.20)	

**Legend for Table 4, Table 5 & Table 6:**

Null model is a model in which all of the factors are uncorrelated. Model 1 General =~ CS+RC+EC+FM+GM.

X2: chi-square, Df: degrees of freedom, AIC: Akaike’s information criterion, BIC: Schwarz’s Bayesian information criterion (adjusted for sample size), CFI: Comparative Fit Index, TLI: Tucker-Lewis Index, RMSEA: Root Mean Square of Approximation, CI: 95% Confidence Interval, MI: Measurement invariance

Non-significant Chi square statistics at p=0.05 level and RMSEA < 0.05 indicate good fit. A confidence interval <0.08 derived from RMSEA was also taken as an indicator of good fit. CFI has acceptable fit at 0.9, and good fit at >0.957. TLI has good fit >0.9. A p-value of 0.05 was taken to be significant in all analyses.

AIC: Lower is better. Attempts to select models that are the most parsimonious/efficient representations of the observed data. BIC is similar to AIC but more conservative.

**Table 5.** General factor model fit by gender in individual age groups.

	Goodness-of-Fit Indices								
Model – Male	X2	Df	X2/Df	p-value	AIC	BIC (Adjusted)	CFI	TLI	RMSEA (CI)
Group 1 (N=47) Age >0<=12 months									
Null Model	218.37	10	21.84	<0.001					
Model 1	6.63	5	1.33	0.25	1106.00	1124.50 (1093.14)	0.99	0.98	0.08 (0.00-0.23)
Model1-FM~~GM	6.63	4	1.66	0.16	1108.00	1128.35 (1093.85)	0.99	0.97	0.12 (0.00-0.27)
Group 2 (N=56) Age >12<=24 months									
Null Model	255.13	10	25.51	<0.001					
Model 1	22.94	5	4.59	<0.001	1522.19	1542.44 (1511.01)	0.93	0.85	0.25 (0.15-0.36)
Model1-EC~~RC	2.59	4	0.65	0.63	1503.84	1526.12 (1491.55)	1.00	1.00	0.00 (0.00-0.17)
Group 3 (N=44) Age 24–43 months									
Model 1	4.53	5	0.91	0.48	1217.50	1235.345 (1204.009)	1.00	1.00	0.00 (0.00-0.20)
Group 1 (N=39) Age >0<=12 months									
Null Model	209.36	10	20.94	<0.001					
Model 1	13.85	5	2.77	0.02	975.49	992.13 (960.85)	0.96	0.91	0.21 (0.08-0.35)
Model1-FM~~GM	5.40	4	1.35	0.25	969.04	987.34 (952.93)	0.99	0.98	0.10 (0.00-0.27)
Group2 (N=54) 12<=24 months									
Null Model	245.56	10	24.56	0.00					
Model 1	14.49	5	2.90	0.01	1448.24	1468.13 (1436.71)	0.96	0.92	0.19 (0.08-0.30)
Model1-EC~~RC	0.73	4	0.18	0.95	1436.48	1458.36 (1423.80)	1.00	1.00	0.00 (0.00-0.02)
Group 3 (N=27) Age >24<=43 months									
Model 1	4.51	5	0.91	0.48	755.32	768.28 (737.21)	1.00	1.00	0.00 (0.00-0.25)

**Table 6.** Nested models in multi-group confirmatory factor analysis by gender.

Gender Invariance								
	Df	X2	P value	X2 diff	Df diff	P value	CFI	ΔCFI
Group 1: Age >0<=12 months Model1-FM~~GM								
**Configural Invariance**	8	12.03	0.15				0.99	
**Metric Invariance**	13	19.93	0.12	7.91	5	0.16	0.98	0.00
**Scalar Invariance**	17	21.02	0.27	1.09	4	0.90	0.99	0.00
**Strict Invariance**	22	28.98	0.14	7.97	5	0.16	0.98	0.01
Group 2: Age >12<=24 months Model1-EC~~RC								
**Configural Invariance**	8	3.32	0.91				1.00	
**Metric Invariance**	13	7.58	0.87	4.26	5	0.51	1.00	0
**Scalar Invariance**	17	9.77	0.91	2.19	4	0.70	1.00	0
**Strict Invariance**	22	12.21	0.95	2.44	5	0.79	1.00	0
Group 3: Age >24<=43 months Model 1								
**Configural Invariance**	10	9.03	0.53				1.00	
**Metric Invariance**	14	14.37	0.42	5.33	4	0.26	1.00	0
**Scalar Invariance**	18	17.29	0.50	2.92	4	0.57	1.00	
**Strict Invariance**	23	24.21	0.39	6.92	5	0.23	1.00	

**Legend for Table 6:**

X2 diff – chi-square difference between models, Df diff; change in degrees of freedom between models. Between nested models, if P value> .01 (insignificant)--the fit of the model has not been significantly hindered by introducing the additional constraints so the increase in χ2 value is not significant in reducing model fit.

ΔCFI – if <0.01 there is not a significant change in model fit between nested models.

Configural Invariance: baseline model to which we can compare more restrictive models. Same common factors across groups

Metric Invariance: Common factors have the same meaning across groups

Scalar Invariance: Group differences in observed means will be directly related to group differences in factor means

Strict Invariance: Group differences in observed means and variances will equal corresponding group differences in factor means and variances

Group 1 (0–12 months): The construct structure with one general factor (Model 1) demonstrated unacceptable model fit, with RMSEA above 0.1 and significant chi square. The modification indices suggested residual correlation between gross and fine motor domains indicating the scores observed in gross and fine motor skills share additional variance that is not explained by the general factor. Once these residuals were allowed to be correlated in the model (Model 1 FM ^~~^GM), the model fit improved significantly (non-significant chi square test, reduction in AIC and BIC) with RMSEA at 0.07 and almost perfect TLI and CFI.

Group 1 by gender: The male group for Model 1 showed acceptable model fit, while the female group fit optimally in the model with residual correlation of gross and fine motor skills. We carried out MG-CFA on the model with residual correlation of motor domains, and established that strict invariance was achieved, as the differences in chi square between nested models were not significant.

Group 2 (12–24 months): The model fit for the one-factor solution (Model 1) was not acceptable for Group 2, as the chi square test was significant, RMSEA was 0.23, and TLI was below 0.9. The modification indices suggested residual correlation between receptive (RC) and expressive (EC) domains would improve the model fit. Using Model 1-EC ^~~^RC, the model fit was greatly improved, (non-significant chi square test, reduction in AIC and BIC). Subsequently, we carried out MG-CFA using the revised model. The results showed that the model fit for all models was very good, and the change in model fit was not significant, indicating that strict invariance was established.

Group 3 (24–43 months): The CFA result using Model 1 demonstrated acceptable model characteristics, with RMSEA lower than 0.1 and CFI and TLI both above 0.95. Therefore, we accepted this model and performed MG-CFA afterwards. Consistently, strict invariance was identified for Group 3, as the change in model fit was consistently non-significant.

MG-CFA on the three groups used the models derived from the CFA analysis. Strict gender invariance was achieved for all groups, with correlated residuals constrained in Groups 1 and 2. In Group 1 and Group 3, no significant difference in latent means could be observed between the two genders. In Group 2, there was a marginally significant (p=0.05) difference in the latent means between genders, suggesting that girls performed better than boys on this tool of general neurodevelopment at the age of 12–24 months.

## Discussion

Viet Nam is the fourteenth most populous country in the world ^38^. Achievements on the Millennium Development Goal targets put the country in a good position to tackle the 2030 Sustainable Development Goals ^39^, for which a reliable and valid CDAT is required to assess needs and track progress.

This study demonstrates that our adaptation of the Bayley III for use in an urban Vietnamese population has good reliability, and also meets strict invariance criteria for gender invariance by age group. However, the structure of the adapted tool differs slightly from the original US version. In our adaptation we identified three different models for the three age groups we evaluated. The changes we made are consistent with early development theories, which suggest that initial skills attained in the first year after birth are primarily motor, followed by language development increasing from the second year of life onwards ^40^. By comparison, a Brazilian adaptation of the Bayley III tested on 207 children aged 12–42 months, found a general factor was the best fit ^41^. This was interpreted as a global measure of child development.

Change in factor structure with age has been demonstrated in other psychological studies. Martins *et al.* evaluated the factorial structure of cognitive abilities in 472 children aged 4–10 years, split into 3 age groups ^42^. Measurement invariance was not met, and Martins concluded ‘ *children’s cognitive abilities and their structure are unstable, thus their emergence could be conditioned by school learning and everyday experiences.*’ ^42^ Similarly, Lee *et al*. identified changes in executive function factor structure with age ^43^, changing from a two-factor structure in early childhood to a three-factor structure among the teenagers in a cohort of 688 children aged 6–15 years.

The original Bayley study used data from a stratified sample of 1700 children from across the US. In contrast, this study focused on a smaller sample of Vietnamese children from an urban district in HCMC, and the socioeconomic details for the participants were significantly different from publicly available census data for the general Vietnamese population. Although this is a clear limitation of the study, the work represents the first attempt to develop a locally relevant adapted tool for Vietnam and to formally evaluate the psychometric properties of the adapted tool. The research paves the way for further work going forward, potentially expanding data gathering to include rural populations and to extend the tool’s applicability more broadly across Vietnam. For the present, this adaptation has both clinical utility and is suitable for use in research studies involving urban Vietnamese children, and should prove to be a valuable instrument for evaluating early child development in this population.

- *“What is already known on this topic”*
  - There is limited published literature on the process of validating Bayley III adaptations.
  - Establishing robust psychometric properties for an adapted child development assessment tool is important to allow meaningful interpretation of data collected using the tool
  - Reported differences in scores between genders on Bayley III may be due to the test having different developmental meaning between genders.
- *“What this study adds”*
  - This study outlines a method of assessing reliability and construct validity of an adapted test.
  - The construct structure of the Vietnamese Bayley III varied by age in keeping with expected child development.
  - The adaptation was not biased by gender and is suitable for use in future studies in urban Vietnamese populations.

## Data availability

### Underlying data

Open Science Framework: Bayley VN. https://doi.org/10.17605/OSF.IO/JXBUQ ^18^

This project contains the following underlying data:

- wide_data_12_6_19.csv (Bayley III results for participants)

### Extended data

Open Science Framework: Bayley VN. https://doi.org/10.17605/OSF.IO/JXBUQ ^18^

This project contains the following extended data:

- Case report form.doc (Study case report form)
- Supplem22_1_19.docx (Document containing example modifications to the Bayley III for Viet Nam and confirmatory factor analysis diagram)

Data are available under the terms of the Creative Commons Attribution 4.0 International license (CC-BY 4.0).
