# Supplementary material for: Bayley III in Vietnamese children: lessons for cross-cultural comparisons
Source: Wellcome Open Res. 2019 Jun 19;4:98. [Version 1] doi: 10.12688/wellcomeopenres.15282.1 (PMC6685399; doi:10.12688/wellcomeopenres.15282.1)
Supplement: Peer review history

# Peer review: Reviewer response for version 1

**DOI:** 10.21956/wellcomeopenres.16680.r35963

**Published:** 2019-08-06

**Authors:** Carme Brun-Gasca

**Affiliations:** 1 Department of Clinical and Health Psychology, Autonomous University of Barcelona, Cerdanyola del Vallès, Spain

This is an interesting paper about the adaptation of the Bayley III, a very useful tool for children assessment, in Vietnam population.

 The authors have followed the appropriate process so that the study can be considered valid and replicable.

 The authors indicate that they have not translated or adapted the adaptive behavior scale but they did not justify this issue, though they said that the company of the original instrument agreed with the decisions made.

 A major point I would suggest is that the research would benefit of a more representative sample in terms of socioeconomic level, and that would probably change some of the results

I confirm that I have read this submission and believe that I have an appropriate level of expertise to confirm that it is of an acceptable scientific standard.

# Peer review: Reviewer response for version 1

**DOI:** 10.21956/wellcomeopenres.16680.r35872

**Published:** 2019-08-05

**Authors:** Daynia Ballot

**Affiliations:** 1 Division of Neonatology, Department of Paediatrics and Child Health, University of the Witwatersrand, Johannesburg, South Africa

This article provides detailed analysis of an adaptation of the Bayley scales of infant development version 3 for use in Vietnam.

 Please provide information on the number of children who declined/you excluded from participation in the study.

 The assessment tool was translated into Vietnamese. Is there only one official language in Vietnam? Many low and middle-income countries have multiple official languages.

 You state that you made other adaptations to the assessment tool – using three models for three different age groups. Please summarise these adaptations for this article.

 I cannot comment adequately on the statistics – which seem to be sound, but I recommend review by a statistician.

I confirm that I have read this submission and believe that I have an appropriate level of expertise to confirm that it is of an acceptable scientific standard.
